# Effectiveness of a tailored implementation strategy to improve adherence to a guideline on mental health problems in occupational health care

**DOI:** 10.1186/s12913-019-4058-5

**Published:** 2019-05-03

**Authors:** Margot C. W. Joosen, Karlijn M. van Beurden, David S. Rebergen, Monique A. J. M. Loo, Berend Terluin, Jaap van Weeghel, Jac J. L. van der Klink, Evelien P. M. Brouwers

**Affiliations:** 1 Tilburg University, Tilburg School of Social and Behavioral Sciences, Tranzo Scientific Center for Care and Wellbeing, Tranzo, Postbus 90153, 5000 LE Tilburg, The Netherlands; 2 Tilburg University, Tilburg School of Social and Behavioral Sciences, Department Human Resource Studies, Tilburg, The Netherlands; 3 Shared Ambition, People Management, Amersfoort, The Netherlands; 4 M.A.J.M. Loo, Epe, The Netherlands; 5 Department of General Practice and Elderly Care Medicine, VU University Medical Center Amsterdam, Amsterdam Public Health research institute, Amsterdam, The Netherlands; 6 Phrenos Centre of Expertise, Utrecht, The Netherlands; 7 Parnassia Group, Dijk en Duin Mental Health Center, Castricum, The Netherlands; 8 Netherlands School of Public & Occupational Health, Utrecht, The Netherlands

**Keywords:** Mental health, Practice guideline, Occupational medicine, Guideline adherence, Implementation, Occupational health professionals, Occupational health, Work disability prevention

## Abstract

**Background:**

As compliance to guidelines is generally low among health care providers, little is known about the impact of guidelines on the quality of delivery of care. To improve adherence to guideline recommendations on mental health problems, an implementation strategy was developed for Dutch occupational physicians (OPs). The aims were 1) to assess adherence to a mental health guideline in occupational health care and 2) to evaluate the effect of a tailored implementation strategy on guideline adherence compared to traditional guideline dissemination.

**Methods:**

An audit of medical records was conducted as part of a larger RCT study. Participants were 66 OPs (32 intervention and 34 control) employed at one of six sites of an Occupational Health Service in southern Netherlands. OPs in the intervention group received multiple-session peer group training which focused on identifying and addressing barriers to using the guideline, using a Plan-Do-Check-Act approach. The control group did not receive training.

Medical records of 114 workers sick-listed with mental health problems were assessed (56 intervention and 58 control). Guideline adherence was determined by auditing the records using 12 guideline-based performance indicators (PI), grouped into 5 PIs: process diagnosis, problem orientation, interventions/treatment, relapse prevention, and continuity of care. Differences in performance rates of the PIs between the intervention and control groups were analyzed, taking into account the cluster study design.

**Results:**

OPs who received the training showed significantly greater adherence compared to the controls (*p* < .028) in 4 out of 5 grouped PIs, i.e. process diagnosis, problem orientation, interventions/treatment and relapse prevention. In one out of 12 PIs adherence was found adequate (53% of the medical records), in 6 PIs adherence was found minimal, and in 5 PIs the majority of the records showed no adherence.

**Conclusions:**

An implementation strategy which addressed key barriers for change and tailor-made interventions improves adherence to an occupational health guideline for mental health problems compared to traditional guideline dissemination. However, adherence to the guideline recommendations is still far from optimal. To optimize adherence, it is recommended that implementation strategies focus on the workers level, organizational level, and the professional level.

**Trial registration:**

ISRCTN86605310. Registered 30 June 2010.

**Electronic supplementary material:**

The online version of this article (10.1186/s12913-019-4058-5) contains supplementary material, which is available to authorized users.

## Background

Adherence to guidelines is generally low among health care professionals, even though many evidence-based practice guidelines exist and are recommended to be used in health care [1, 2]. Lack of guideline adherence can lead to refraining from essential care, suboptimal patient outcomes and wasted resources [3]. To improve the quality of patient treatment and decrease variability in care, it is important to improve implementation of and adherence to practice guidelines [4].

Unfortunately, many studies have demonstrated a lack of compliance to guidelines [5, 6]. As a result, it remains unclear whether practice guidelines have any impact on the performance of the providers and on outcomes on patient level [7]. Multiple factors can be of influence on guideline implementation, such as patient and provider characteristics, environmental factors, and the socio-political context [6, 8]. Cabana and colleagues [9] have shown that barriers to guideline adherence can be prevalent on different levels; barriers might be knowledge-related like a lack of awareness with the guideline, or attitude-related such as a lack of agreement with specific recommendations, lack of self-efficacy and skills to apply recommendations, or lack of motivation to change physician routine. Also external barriers can obstruct guideline use, such as patient factors (i.e. preferences of patients), guideline factors (i.e. not clearly written), and environmental factors like lack of time or resource, or organizational constraints hindering guideline use. To improve guideline adherence, passive implementation strategies, such as guideline dissemination, are found to be ineffective. Also, education meetings that solely focus on improving knowledge (such as lectures) will not lead to the desirable change in behavior of professionals [10]. To be effective in improving adherence, active strategies are needed that aim to eliminate barriers that hinder professionals from adhering to a specific guideline [11]. Therefore, firstly it is important to identify the barriers that are perceived by the target group [12]. Furthermore, it is recommended to use implementation strategies tailored to the needs of the target group to overcome perceived barriers of specific guideline recommendations [11, 13].

In occupational health care, scientific evidence is growing and practice guidelines are increasingly developed for their use in occupational health care settings [14–16]. Specifically for the treatment of mental health problems by occupational health professionals, various practice guidelines have been developed worldwide [17–19]. The Dutch guideline entitled ‘The management of mental health problems of workers by occupational physicians (OPs)’ (further referred to as ‘MHP guideline’) is one of these [20, 21]. Currently, work disability is primarily caused by mental health problems, such as mood and anxiety disorders and provokes major challenges for societies all over the world [22–24]. The MHP guideline aims to establish improved and sustainable work functioning and relapse prevention among workers with mental health problems. One of the central aspects of this guideline for OPs is to follow an activating approach aiming to enhance the problem solving capacity of workers, particularly in relation to their work context. Despite various efforts to implement the MHP guideline, research shows that OPs only minimally adhere to the guideline’s recommendations in practice [25, 26]. However, OPs do have a positive attitude towards the guideline in general and they have the intention to use it [27].

This study is part of a larger project which aims to explore how the management of sick-listed workers with mental health problems by OPs can be improved [28]. In the larger project, an implementation strategy (i.e. interactive peer group training) is developed, implemented and tested to improve guideline adherence among OPs, and to evaluate its effect on workers’ return to work using a cluster randomized controlled trial [28]. In a qualitative study as part of the larger project, it was found that OPs perceived multiple barriers to use the guideline in their practice, including a lack of knowledge of the content of all guideline parts and attitude related barriers such as lack of self-efficacy to perform certain recommendations, and difficulties with changing their routines and habits [29]. Additionally, external barriers, most commonly work contextual barriers, obstructed guideline use. These were a lack of time and perceived work pressure, and tight contracts between employers and occupational health services in which for example agreements are made about the frequency of consultations. Also a lack of collaboration with other stakeholders such as the employer and other health care providers, and conflicting policy was perceived as a barrier to adequately use the guideline as recommended [29]. In a process evaluation, it was found that the peer group training was a feasible, and a highly valued method among participating OPs. The training added to OPs’ knowledge, attitudes and skills, but OPs still perceived various external barriers using the guideline [30]. When assessing workers’ outcomes, this revealed that the guideline implementation approach did not lead to shortened sickness duration in workers with mental health problems [*n* = 3228] [31]. Additional analyses on the association between the use of the guideline by OPs and workers’ outcome, found that better guideline use was not associated with earlier return to work [32]. From these previous studies we know that the implementation strategy was not effective on the workers’ level, i.e. did not lead to shorter duration of return to work. On the OPs’ level, we found that OPs were highly satisfied with the guideline training and self-perceived adherence was high. However it is unknown to what extend OPs are actually using the guideline in practice. The current study aims to assess adherence to the MHP guideline by OPs and the effect of a tailored implementation strategy on guideline adherence in addition to implementation as usual (that is, dissemination of the guideline among Dutch OPs and providing short continuing medical education courses) by means of an audit of medical records.

Research questions:

1. To what extent do occupational physicians (both intervention group and control group) adhere to the MHP guideline?
2. What is the effect of a tailored implementation strategy on guideline adherence among occupational physicians compared to implementation as usual?

## Method

### Study design

The current study was part of a larger project, examining the effect of an intervention to enhance guideline adherence in OPs on return to work of workers with mental health problems [28]. In the larger project workers with mental health problems were included and received guidance by an OP who also participated. Randomization to the control and intervention group was performed at the level of participating OPs. The OPs in the intervention group received an innovative peer group guideline training and received educational credits after completing the training. OPs in the control group received no extra training in the guideline and performed care as usual. In the years previous to this study, the guideline was distributed among Dutch OPs and became part of their continuing medical education. Therefore, it is assumed that most of the OPs had at least minimal knowledge of the guideline.

After the training, data on guideline adherence were collected by means of an audit of medical records of sick-listed workers with mental health problems who were participating in the larger project, and who were guided by an OP in the in the intervention or control condition. These data were collected between November 2012 and January 2014. The current study reports on the data analysis of the audit of medical records.

Approval was obtained from the Medical Research Ethics Committee of St. Elisabeth Hospital in Tilburg. This study was registered in the ISTCTN trial register, ISRCTN86605310. The “CONSORT 2010 statement: extension to cluster randomized controlled trials” was used for reporting [33].

### Setting

In the Netherlands, according to the Dutch Gatekeeper Improvement Act [34, 35], the employer is responsible for the return to work of sick-listed workers during the first 2 years of sickness absence. During this period the employer is obliged by law to continue paying wages (at least 70%). This is irrespective of cause and work-relatedness. During these 2 years the sick-listed worker cannot be fired because of his sickness.

The OP has a central role in the Dutch social security system, and is the link between workers’ health and the work situation. An OP is a qualified medical doctor specialized in occupational health who assists employers and workers in occupational health issues, safety and sickness absence management [34]. In the case of sickness absence of a worker, the employer is obligated by law to provide access to an OP within 6 weeks of the sickness absence. The OP provides return to work support to the sick-listed worker and provides advice to the employer regarding return to work activities and work adaptations if necessary. Most Dutch employers have contracted independently operating OPs or Occupational Health Services (OHSs) for these services. Within these contracts employers and the OHS or OP agree on the tasks for and conditions under which the OPs can operate, including the frequency and, available time for consultations with the worker, as well as providing access to work sites. OPs often work for several organizations (ranging from large companies to small businesses) at multiple locations.

Since 1998, The Netherlands Society of Occupational Physicians (NVAB), has developed and implemented evidence-based guidelines for OPs for a variety of health conditions [36]. In this study we used the MHP guideline for OPs, which was developed in 2000 [20] and revised in 2007 [21]. The guideline is of ‘moderate to high developing and reporting quality’ according to appraisal using the internationally validated AGREEII instrument [19].

The MHP guideline [21] recommends OPs to use a process-based approach in both case and care management, and to document their findings in the workers’ medical record. The content of the guideline is based on cognitive behavioral principles aiming to enhance the problem solving capacity of both workers and employers, particularly in relation to the work context (See Table 1). Depending on the needs of the worker, the OP can choose to provide care management in addition to case management. Most workers will be receiving care from their general physician as well. Additionally, the OP can refer the worker to specialized (mental) health care. It is recommended that the OP communicate with other health care providers to try to align the treatments and promote healthy functioning and the value of work in addition to medical care. With regard to the employer, the OP advises and supports the employer/supervisor on the return to work process and any necessary work adaptation to achieve sustainable participation. This can be done through a wide variety of means (e.g. email contact, face-to-face meetings).

**Table 1:** Summary of the content of the MHP guideline [21, 29, 30]

Part of the guideline	Content and recommendations
1. Problem Orientation and Diagnosis	An early involvement of the OP in the sick leave process of the worker is promoted (first consultation within 2 weeks after the worker reports sick). A simplified classification of mental health problems is introduced in four categories: i) stress-related complaints, ii) depression, iii) anxiety disorder, and iv) other psychiatric disorders. Furthermore, the problem inventory should focus on factors related to the worker, his or her work environment, and the interaction between these two.
2. Intervention/Treatment	The OP acts as case manager by monitoring and evaluating the recovery process. If recovery stagnates, the OP should intervene by acting as care manager by using cognitive behavioral techniques to enhance the problem-solving capacity of the worker, providing the worker and the work environment with information and advice on the recovery and the RTW process, contact the general practitioner when problems remain the same or increase, and refer the worker to a specialized intervention if necessary. In addition, the OP should advise the work environment (e.g., supervisors, managers, and human resource managers) on how to support the worker and enhance the recovery and RTW process.
3. Relapse Prevention	Integration of relapse prevention from the first contact with the worker by enhancing the problem-solving capacity of the worker. The newly acquired problem solving skills are explicitly addressed in at least one specific relapse prevention meeting after RTW.
4. Continuity of care / Evaluation	During all meetings, evaluation of the recovery process includes the perspectives of the worker, supervisor, and other involved professionals. Follow-up meetings with the worker should take place every 3 weeks during the first 3 months, and every 6 weeks thereafter. The supervisor or work environment should be contacted once a month. Follow-up contacts with the general practitioner or other professionals should take place when the recovery process stagnates or when there is doubt about the diagnosis or treatment.

Source: NVAB guideline *‘*The management of mental health problems of workers by occupational physicians’ [21], see also [29, 30]

*OP* Occupational physician, *RTW* Return-to-work

### Implementation strategy – intervention group

The peer group training for OPs was developed as a tailored implementation strategy aimed at improving OPs’ adherence to the MHP guideline. The training consisted of eight two-hour-meetings which were scheduled over the course of 1 year; January 2011 – January 2012. The training sessions were held at six regional offices of the OHS across the southern part of the Netherlands; each group attended the training at one location.

Small interactive groups of four to six OPs were formed to stimulate involvement and encourage in-depth discussion among OPs and to learn from their peers. The trainer (MJ) guided the groups by structuring the meetings, facilitating the discussions and monitoring the progress. The training focused on identifying and addressing barriers OPs perceived in using the guideline recommendations in practice. The framework of Cabana was used to identify barriers related to knowledge, attitude and external factors [9]. The training incorporated the different parts of the guideline, with each session focusing on a different topic and guideline recommendations. A Plan-Do-Check-Act (PDCA) approach was used by the trainer to structure and moderate the discussions; explore barriers perceived by OPs, find suitable solutions to overcome these barriers, test these solution in practice and evaluate the results. The PDCA cycle follows a learning approach aiming to change behavior and is flexible in adapting the changes according to feedback, which helps to ensure that fit-to-purpose solutions are developed [37]. The focus on perceived barriers (i.e. the Cabana model) in combination with a PDCA approach formed the basis of the training on the MHP guideline (see Table 2).

**Table 2:** Structure of the guideline training ‘Mental Health Problems’ [30]

Structure (Plan-Do-Check-Act)	Explanation
Stepwise discussion of the guideline content (Plan1)	In each meeting, the recommendations of part of the guideline are discussed
Barrier analysis: knowledge, attitude, and external barriers (Plan2)	Identify individual and group barriers that hinder OPs from using the guideline by discussing guideline recommendations (a different part of the guideline in each meeting)
Discussion of possible solutions for specific barriers (Plan3)	OPs discuss how specific barriers can be overcome by suggesting solutions to apply in practice
Action plan (Plan4)	OPs draw up an action plan of how to implement these solutions in their daily practice, and agree on learning objectives and ‘homework’ assignments
Practice of suggested solutions (Do)	OPs test the suggested solutions to experience how and if these would help in applying the guideline recommendation
Evaluation of experiences (Check)	OPs’ experiences with the suggested solutions are evaluated to decide what did work and what did not work for performing the guideline recommendation
Adjustment of solutions if necessary (Act)	If necessary, the solutions are adjusted according to what OPs experience in practice

Source: Joosen et al. 2015 [30]

*OP* Occupational physician

In each session the trainer (MJ) asked the OPs to discuss which barriers they perceived in adhering to that specific topic. Next, OPs suggested solutions to address these barriers, taking into account the context of their daily practice. Subsequently, OPs tested the solutions in their daily practice. Finally, results were evaluated and, if necessary, solutions were adjusted. This PDCA cycle was repeated in each meeting and for all topics stated in the guideline. An additional file illustrates how OPs were engaged in the implementation of the guideline recommendations and how a PDCA cycle was applied (See Additional file 1).

In a previous reported study, Joosen and colleagues [30] reported on the compliance and feasibility of the peer group training, showing that the protocol was carried out as planned, all participating OPs attended all 8 training sessions and 90% of the OPs agreed that the peer-learning groups and the meetings spread over 1 year were highly effective training components. A detailed description of the implementation strategy and its feasibility can be found elsewhere [30].

### Participants

#### Occupational physicians

OPs were recruited from a large OHS in the Netherlands between October 2010 and January 2011. All OPs who were employed at one of six sites of the OHS in the southern part of the Netherlands (n = approximately 155) were invited to participate. First, presentations by the researchers (MJ and EB) and information about the larger project were provided at several meetings for OPs at the OHS, after which OPs could register for participation. Also, an email invitation was sent to all OPs; a reminder email was sent after 2 weeks, and all OPs who had not yet responded were contacted by telephone.

#### Medical records

Medical records of sick-listed workers who were guided by participating OPs were used to assess adherence. Workers were selected from the registration system of the OHS based on the following inclusion criteria: 1) CMD was the primary reason for sick leave diagnosed by an OP according to the Dutch Classification of Diseases, based on the ICD-10 [38], 2) being on current sick leave when selected from the registration system of the OHS after the first meeting with the OP (between January 1st 2012 and January 15th 2013), and 3) having adequate command of the Dutch language. Exclusion criteria were: being suicidal, and a physical problem being the primary reason for sick leave at the time of study inclusion. The OHS invited the eligible workers to participate in this study. Participating workers gave their written informed consent and signed a separate consent form when they gave permission to audit their medical records. The audited period was 12 months, starting the first day of sickness absence of each worker. After inclusion, questionnaires were filled out regarding socio-demographic characteristics (age, gender, educational level), personal and work factors (contract hours and workability measured with a single question of the workability index (WAI) [39]) and clinical characteristics (measured with the Four-Dimensional Symptom Questionnaire (4-DSQ) [40]). More details about these questionnaires are described elsewhere [28].

### Randomization, stratification and blinding

In each of the six regional offices of the participating OHS, a peer training group of OPs (intervention group) and a control group was formed. Randomization took place on the level of OP by computerized allocation. To establish equal intervention and control groups at all six sites, pre-stratification was used and OPs working at each site were randomly allocated to one of the two groups. To limit the risk of contamination across OPs working at the same site we specifically asked the OPs in the intervention group not to discuss the content of the guideline training.

OPs were not informed about the inclusion of specific workers into the study, but they were told which workers were invited to participate (about 500 workers in total). Workers and employers were blinded for randomization since they were not aware of the group allocation of their OP. During the recruitment, intervention period and data collection researchers were not blinded. During data analysis, the researchers were blinded for allocation of the groups since information related to the OP were stripped from the data.

### Data collection on guideline adherence

#### Performance indicators (PIs)

Adherence to the guideline was determined by assessing medical records in which OPs record the provided care. In previous studies a set of performance indicators (PIs) was used to evaluate adherence to the MHP guideline developed in 2000 [25, 26, 41]. As the guideline was revised in 2007 the initial PIs did not fully cover the content of the revised guideline. Therefore, a new set of PIs was developed that covered the essence of the revised guideline.

The set of PIs was systematically developed using an iterative consensus rating procedure in three steps: (i) preselection of recommendations; (ii) expert consensus procedure; and (iii) transcription and classification of final set of indicators [42–44].

1. Preselection of recommendations. Three authors (JvdK, DR, ML) independently preselected all recommendations from the MHP guideline. They focused on the most important guideline recommendations that would have impact on the quality of occupational health care. This resulted in a list of 24 recommendations.
2. Expert consensus procedure. An expert meeting was organized with mental health specialists, work and health specialists, quality of care researchers, OPs, an occupational therapist, a general practitioner and a patient representative. In a two-round consensus procedure, the panel of 9 experts discussed the relevance of the recommendations to physicians’ performance and patients’ health benefit. Moreover, the experts discussed which recommendations reflected the essence of the guideline and how the PIs should be best formulated. This resulted in a selection of 20 recommendations.
3. Transcription, pilot testing and classification of final set of indicators. The selected 20 recommendations were transcribed into indicators and a subsequent scoring set. Ten medical records were pilot tested by two researchers independently. Based on the comments of the reviewers about feasibility (e.g. usability), measurability (e.g. is all information needed available in medical records) and relevance (e.g. are indicators relevant to quality of care), the list of PIs was adjusted. The final set of PI’s consisted of 12 indicators grouped into 5 categories of indicators (further referred to as grouped PIs). The PIs measured different aspects of the management of mental health problems, including diagnosis, management of mental health problems, relapse prevention and continuity of care. The PIs are presented in Table 3.

**Table 3:** Description of 12 PIs for OP’s guideline adherence in workers’ medical records and criteria for their scoring [31]

PI		Criteria	Scoring^a^
1. Process diagnosis			
1.1	Monitoring the recovery phase of the worker	The process of recovery (i.e. phase of the recovery process: crisis phase, problem solving phase, implementation phase) should be monitored throughout the sickness absence period	0 = Recovery phase not documented 1 = Recovery phase occasionally documented 2 = Recovery phase regularly documented
1.2	Assessment of the worker’s recovery tasks	The tasks needed to achieve recovery should be assessed throughout the sickness absence period (e.g. gaining insight into what happened, accepting the situation, regain day structure, problem identification and finding solutions, implement solutions, regain roles)	0 = Recovery tasks not documented 1 = Recovery tasks occasionally documented 2 = Recovery tasks regularly documented
1.3	Assessment of the employers’ perspective	The way the employer (e.g. supervisor, management, human resource management) copes with the sick-listed worker and their perspective on recovery should be assessed during the sickness absence period	0 = No information about employers’ perspective 1 = Occasional information about employers’ perspective 2 = Clear description of the employers’ perspective in relation to the worker’s situation
2. Problem orientation			
2.1	Problem identification	The relation between factors that influence the mental health problems and performance at work and home should be identified (e.g. overburdened by high workload or work conflict or lack of social support)	0 = Problems not documented 1 = Problems documented, relation with performance not documented 2 = Problems and their relation with performance documented
2.2	Assessment of symptoms	Presence or absence of essential symptoms of mental health problems should be assessed (i.e. distress, depression, anxiety, and somatization)	0 = No symptoms documented 1 = Symptoms occasionally documented 2 = Presence or absence of the essential symptoms documented
2.3	Diagnosis	Diagnosis based on ICD-10 and supported with arguments	0 = No diagnosis documented 1 = Diagnosis documented without arguments 2 = Diagnosis documented, including arguments
3. Intervention/Treatment			
3.1	Evaluation of the worker’s course of the recovery process	The course of the recovery process (stagnation or recovery process as expected) should be evaluated and supported with arguments.	0 = Course of recovery process not documented 1 = Course of recovery process documented without arguments 2 = Course of recovery process documented including arguments
3.2	Treatment in accordance with the worker’s recovery process	IF recovery process is ‘as expected’ the OP acts as process manager by monitoring the process of recovery and using minimal interventions. IF recovery process stagnates the OP also acts as care manager by providing a more extensive guidance with treatment based on cognitive behavioral techniques, providing the employer with advice on recovery and the RTW process, contacting other health care professionals (e.g. general practitioner, psychologist), and if necessary referring the worker to specialized care.	0 = Treatment is not in accordance with the recovery process 1 = Treatment is in accordance with the recovery process without argumentation 2 = Treatment is in accordance with the recovery process including argumentation
4. Relapse prevention			
4.1	Relapse prevention	Relapse prevention should be integrated during consultations AND the OP has at least one consultation with the worker after full RTW	0 = No information on relapse prevention documented 1 = Information on relapse prevention during or after the sickness absence period documented 2 = Information on relapse prevention during the sickness absence period documented AND OP had at least one consultation with the worker after full RTW
5. Continuity of care/Evaluation			
5.1	Rapid first consultation	First face-to-face consultation within 15 days from the 1st day of sickness absence.	0 = First consultation after 22 days 1 = First consultation between 15 and 22 days 2 = First consultation within 15 days
5.2	Regular contact with the worker	Consultations with the worker take place every 3 weeks during the first 3 months of sickness absence. Thereafter consultations take place every 6 weeks.	0 = Interval between consultations 6 weeks or more during first 3 months AND 9 weeks or more thereafter 1 = Interval between consultations 4–5 weeks during first 3 months AND 7–8 weeks thereafter 2 = Interval between consultations less than 4 weeks during first 3 months AND less than 7 weeks thereafter
5.3	Regular contact with the employer	OP contacts the employer (e.g. supervisor, manager, human resource manager) during the sickness absence period every 4 weeks.	0 = Contacts every 8 weeks or more 1 = Contacts every 5–8 weeks 2 = Contacts every 4 weeks or less

Source: Van Beurden et al., 2018 [32]

*PI* Performance indicator, *RTW* Return to work, *OP* Occupational physician

^a^Scoring: 0 = no adherence, 1 = minimal adherence, 2 = adequate adherence

#### Audit of medical records

From each record, we used the recordings of all consultations from the first day of sick leave until the involvement of the OP ended, or after 1 year sickness absence. Each record was assessed by two assessors independently. The assessors used an audit form which included detailed description of the PIs and instructions for rating each PI. If the rating was not congruent, the two assessors would discuss the case. If no consensus was reached a third assessor audited the medical record and decided about the final score. To guarantee blinding of the outcome assessors, medical records were stripped of information relating to the OP (name, allocation to intervention or control group).

Each of the 12 PIs was rated as 0 (no adherence), 1 (minimal adherence), or 2 (adequate adherence). For each of the five grouped PIs a sum score was calculated by summing the scores of the corresponding PIs divided by the number of PIs. Post hoc, the performance scores were dichotomized because there were too few medical records showing adequate adherence (score 2), see Table 4. Scores were dichotomized into ‘minimal-to-adequate guideline adherence’ (scores ≥1) and ‘no guideline adherence’ (scores < 1). Finally, performance rates were calculated as the percentage of medical records in which guideline-based care was provided.

**Table 4:** Worker’s characteristics in the intervention group and control group

Worker characteristics	Intervention group				Control group			
	n	mean	SD	%	n	mean	SD	%
Age (years)	56	46.1	10.6		58	46.6	10.9	
Gender (male)	22			39.3	25			43.1
Education level								
Low education	6			10.7	2			3.4
Middle-level education	16			28.6	15			25.9
High education	34			60.7	41			70.7
Work and personal related factors								
Working contract hours a week	56	30.5	9.2		58	30.2	10.9	
Workability^a^ (range 0–10)^b^	50	5.3	2.2		53	5.5	2.7	
Clinical characteristics								
Four-Dimensional Symptom Questionnaire (4DSQ) [40]								
Distress (range 0–32)^b^	54	18.1	9.1		55	17.9	9.6	
Depression (range 0–12)^b^	54	2.9	3.7		57	2.7	3.7	
Anxiety (range 0–24)^b^	54	5.2	5.0		55	5.6	5.6	
Somatization (range 0–32)^b^	53	9.2	6.0		54	9.4	7.3	

^a^Measured with the single question of the workability index (WAI) [39]

^b^Higher scores indicate a greater presence of the named factor

### Statistical analysis

To describe guideline adherence among the total group of OPs, descriptive statistics were used among all 12 PIs describing the frequencies of scores on no adherence, minimal adherence and adequate adherence of the guideline.

To evaluate the differences in guideline adherence between intervention group and control group, for each PI the performance rates were compared. We first checked whether it was necessary to control for the cluster level effect of the OPs (i.e. clustering of workers within OPs), using Logistic multilevel analyses within Generalized Linear Mixed Models (GLMM). For all variables the best-fitting model, with or without correction for the cluster level, was chosen based on the Akaike information criterion (smallest AIC represents best-fitting model). If the model with correction for the cluster level was the best fitting model, GLMM analyses were performed. Chi-square analyses were performed if the model without correction for the levels was best fitting. In addition, effect sizes (risk differences) were calculated. The intention-to-treat principle was used in the analyses.

All analyses were performed with SPSS version 19.0.

## Results

A total of 66 OPs agreed to participate and were randomized to the intervention group (*N* = 32) or the control group (*N* = 34). All 32 OPs from the intervention group attended the eight training meetings, in six groups of 4–6 OPs. In six cases an OP was not able to attend a meeting and joined another group for that specific meeting. During the 1 year training period, 10 OPs left their job at the OHS (due to reorganization within the OHS or other reasons) thereby leaving the study. Of the remaining 56 OPs, 26 were in the intervention group and 30 in the control group.

In the larger project, 116 out of 128 workers gave their written consent for auditing their medical record. Two workers were not included in this study; one record was not available at the OHS and in another case mental health problems were not the primary cause for the sickness absence. Therefore, data from 114 workers were used for this study. The included workers were guided by 34 different OPs, 16 in the intervention group and 18 in the control group. From the remaining 22 OPs in this study, no medical records were assessed because the workers in their caseload were not participating in the larger project. Workers’ characteristics are shown in Table 4 and there were no statistically significant differences between workers in the intervention group and control group.

In 109 records the two assessors agreed on the ratings of the PIs. In five records a third assessor was consulted to reach consensus. For the analysis, Chi-square analyses were performed since the model without correction for the cluster levels was best fitting.

### Guideline adherence among all OPs

As can be seen in Table 5, guideline adherence was found to be minimal in 6 out of 12 PIs. In another 5 PIs the majority of medical records showed no adherence. Guideline adherence was especially low in PI 4.1 ‘Relapse prevention by OP’ and PI 5.3 ‘Regular contact employer’ (in respectively 79.8 and 78.9% of the records guideline-based care was not provided). Adequate guideline adherence was found in PI 5.1 ‘Rapid first consultation worker’ (in 52.6% of the records guideline-based care was optimally provided).

**Table 5:** Guideline adherence in medical records (*n* = 114) of OPs in both intervention and control group. Number of medical records in which guideline-based care was not provided (no adherence), minimally provided (minimal adherence) or optimally provided (adequate adherence) and their percentage score (performance rate)

Performance indicator	No adherence n (%)	Minimal adherence n (%)	Adequate adherence n (%)
Process diagnosis			
1.1 Monitoring recovery phase worker	65 (57.0%)^a^	43 (37.7%)	6 (5.3%)
1.2 Assessment of worker’s recovery tasks	52 (45.6%)	59 (51.8%)^a^	3 (2.6%)
1.3 Assessment of the employers’ perspective	38 (33.3%)	58 (50.9%)^a^	18 (15.8%)
Problem orientation			
2.1 Problem identification	5 (4.4%)	88 (77.2%)^a^	21 (18.4%)
2.2 Assessment of symptoms	75 (65.8%)^a^	32 (28.1%)	7 (6.1%)
2.3 Diagnosis	18 (15.8%)	88 (77.2%)^a^	8 (7.0%)
Interventions/treatment			
3.1 Evaluation of the worker’s course of the recovery process	51 (44.7%)	54 (47.4%)^a^	9 (7.9%)
3.2 Treatment in accordance with the worker’s recovery process	56 (49.1%)^a^	44 (38.6%)	14 (12.3%)
Relapse prevention			
4.1 Relapse prevention	91 (79.8%)^a^	21 (18.4%)	2 (1.8%)
Continuity of care			
5.1 Rapid first consultation	36 (31.6%)	18 (15.8%)	60 (52.6%)^a^
5.2 Regular contact with the worker	41 (36.0%)	43 (37.7%)^a^	30 (26.3%)
5.3 Regular contact with the employer	90 (78.9%)^a^	10 (8.8%)	14 (12.3%)

^a^Highest number of medical records within this performance indicator

### Effect of a guideline training on guideline adherence

Table 6 shows the guideline adherence per PI in percentage (performance rate) for both the intervention and the control group. A significantly higher performance rate was found in the intervention group in 4 out of 5 grouped PIs: Process diagnosis (*p* = .011), Problem orientation (*p* = .015), Intervention/treatment (*p* = .015) and Relapse prevention (*p* = .028). No significant differences were found between the groups in grouped PI5 ‘continuity of care’.

**Table 6:** Differences in minimal-to-adequate guideline adherence between intervention and control group. Number of medical records in which guideline-based care was minimal-to-adequate (score 1 and 2) consistent with the guideline, their percentage scores (performance rate) and differences (*p*-value, risk differences and 95% confidence interval) between intervention group and control group (chi-square test)

Performance indicator	Intervention group (*n* = 56)		Control group (*n* = 58)		*P*-value (Pearson Chi-square)	Risk difference (%), 95% CI
	N	%	N	%		
PI1 Process diagnosis	24	42.9	12	20.7	.011*	22.2% [5.5, 38.8]
1.1 Monitoring recovery phase worker	32	57.1	17	29.3	.003*	27.8% [10.4, 45.3]
1.2 Assessment of worker’s recovery tasks	40	71.4	22	37.9	<.001*	33.5% [16.3, 50.7]
1.3 Assessment of the employers’ perspective	37	66.1	39	67.2	.895	−1.1% [−18.5, 16.1]
PI2 Problem orientation	30	53.6	18	31.0	.015*	22.5% [4.9, 40.2]
2.1 Problem identification	56	100.0	53	91.4	.025*	8.6% [1.4, 15.8]
2.2 Assessment of symptoms	24	42.9	15	25.9	.056	17.0% [−0.2, 34.2]
2.3 Diagnosis	50	89.3	46	79.3	.144	10.0% [−3.2, 23.2]
PI3 Interventions/treatment	30	53.6	18	31.0	.015*	22.5% [4.9, 40.2]
3.1 Evaluation of the worker’s course of the recovery process	39	69.6	24	41.4	.002*	28.3% [10.8, 45.8]
3.2 Treatment in accordance with the worker’s recovery process	34	60.7	24	41.4	.039*	19.3% [1.3, 37.3]
PI4 Relapse prevention						
4.1 Relapse prevention	16	28.6	7	12.1	.028*	16.5% [2.0, 31.0]
PI5 Continuity of care	23	41.1	30	51.7	.254	−10.7% [−28.9, 7.6]
5.1 Rapid first consultation	36	64.3	42	72.4	.351	−8.1% [−25.2, 8.9]
5.2 Regular contact with the worker	36	64.3	37	63.8	.956	0.5% [−17.1, 18.1]
5.3 Regular contact with the employer	12	21.4	12	20.7	.923	0.7% [−14.2, 15.7]

*OP* Occupational physician, *PI* Performance indicator

* Significant difference *p* < .05

In 6 out of 12 of the individual PIs the performance rates of the intervention group were significantly higher than in the control group (*p* < .05). Low effect sizes (risk differences) were found between the differences of all PIs (< 27.8%).

## Discussion

In this study we found that OPs who received a tailored guideline training showed significantly greater adherence rates to the guideline for mental health problems in occupational health care compared to OPs who were exposed to traditional guideline dissemination. However, in both groups documented guideline adherence was low. Especially, OPs did not record that relapse prevention was addressed and they did not have regular contact with the employer. Also, symptoms of mental health problems were not documented well and in almost half of the records treatment was not in accordance with the recovery process of the worker. OPs did identify the problems that workers face at work and at home and in most records a rapid first consultation was recorded (within 2 weeks after the 1st day of sick leave).

Overall we found that guideline adherence was poor; in only one PI adherence was found adequate in the majority of the medical records assessed. In previous studies [25, 26], adherence to the MHP guideline was also found to be suboptimal. Although the results cannot be compared on the level of PIs, because the revised version of the guideline with a different content and different set of PIs was used, it is evident that the uptake of the guideline has been problematic for several years.

Several explanations can be given for why we found low guideline adherence. First, an audit of medical records does not reveal what actually happens during the encounters between OP and worker. OPs might not register all their findings and activities in the record. Negative findings and routine activities may not have been documented systematically, with the exception of the frequency of contacts between OP and worker and employer (i.e. continuity of care). Here, the OHS routinely lists the date of each consultation which rules out the possibility of inaccurate registration. Also, the PI criteria were developed to reflect the content of the guideline, but they might not adequately reflect what OPs perceive to be important and relevant to report. Secondly, many employers contract a minimum of services from their OHS (including service by OP). As is shown in the previous reported qualitative study [29], these minimal contracts can be in conflict with guideline recommendations and obstruct OPs from adhering to some recommendations [29]. For example, PI 4.1 ‘relapse prevention’ had one of the lowest performance rates. Possibly in some cases, OPs were restricted in scheduling a relapse prevention consultation because the contract did not cover consultations after full return to work. Besides these organizational constraints, OPs themselves also might not have made optimal use of their position to provide high quality occupational health care. From the analyses of the medical records low performance rates were found on treatment and guidance (PI 3.2). Here, OPs did not act in accordance with the recovery process of the worker (i.e. not intervening when recovery stagnates). Particularly in more complex situations or in case of stagnation the data in the medical records suggested that OPs failed to act as a proactive case manager, e.g. interact with the worker, work system, and other care providers. Especially in these cases acting according to the guideline might result in better worker outcomes.

In a previous outcome study [32], part of the larger project, the relationship between guideline use and workers’ outcomes was investigated using sickness absence registration data from the OHS. The analysis showed that low overall guideline adherence was not associated with earlier return to work. However, when evaluating specific items of the guideline, it was found that regular contact between the OP and the employer was associated with earlier full return to work of workers, even when OPs only minimally adhered to the guideline recommendation [32]. This finding stresses the importance of collaboration between work environment and occupational health professionals in facilitating the return to work process of workers with mental health problems. However, since overall guideline adherence was so poor, it was not possible to evaluate the effect of adequate guideline adherence on return to work. This still leaves the question unanswered whether good guideline use is positively associated with return to work and other workers’ outcomes (such as work functioning or sustainable employability). Meanwhile, it seems important to find strategies to improve adherence to guidelines and at the same time invest in developing/updating guidelines that include high quality evidence and take into account the daily practice and barriers of the target group.

Although overall guideline adherence was poor, we did find that the guideline training resulted in a statistically significant improvement in professional behavior. OPs who received the training reported stronger guideline adherence compared to their colleagues who had not received the training. In a previous study of Rebergen and colleagues [25], no effect of a three-day educational guideline course was found. The additional effect of the current intervention above traditional dissemination and education efforts might be explained by various elements: 1) a peer-group training was used which is known to activate the pre-knowledge of participants, leads to high-quality learning groups, and can impart sustainable knowledge and performance change [45, 46]. In addition, people adopt new information better through their trusted social networks [47]; 2) the training was a participant-focused programme, focusing on barriers OPs perceived in their daily practice (knowledge-related, attitude-related and external barriers), which ensured covering relevant clinical and practical topics. In addition, OPs themselves developed solutions that were tailored to the needs of the OPs and tested the solutions in practice using a Plan-Do-Check-Act approach [37]; 3) The 8 training sessions were spread over the course of 1 year, improving knowledge and allowing OPs to adopt a new working style and actually change their behavior. By this approach, all participating OP were actively involved and felt engaged because they could decide and act on the topics that were most relevant for their ability to use the guideline in their daily practice. Using tailored implementation strategies in small interactive sessions is found to be effective in changing professional behaviour in other studies [48–50].

As part of the larger project, qualitative analyses on the barriers OPs perceive using the guideline were conducted and reported by Lugtenberg and colleagues [29]. For the analyses, the Cabana framework was used to structure barriers into knowledge related barriers, attitude related barriers and external barriers that can influence guideline adherence [9]. It was found that the training had the most impact on knowledge related and attitude related barriers, such as lack of outcome expectancy and lack of self-efficacy, but external barriers remained [30]. The perceived external barriers were mostly work-contextual constraints, such as a lack of time, minimal contracts between OHSs and employers, and conflicting policies of and a lack of collaboration with other stakeholders involved (e.g. employer, healthcare providers) [29]. These kind of external barriers are be too extensive and complex to be changed by a professional-directed intervention [30]. For example, for an individual OP it is difficult to change policy or influence the conditions of contracts as these contracts are usually made between employers and the management of the OHS without interference of the OP. This might explain why we did not find an effect on Continuity of care (PI5), which involves the start of the first consultation, intervals between consultations and contact between OP and employer. Even though the OP *knows* what to do and *wants* to perform a certain behavior, remaining external barriers may prevent actual adherence to all guideline recommendations.

Lack of involvement of different stakeholders, such as the management OHS, employers and other health care professionals during the training might be another reason why OPs were not able to address external barriers. Therefore, to improve the implementation strategy, it would be advised to involve stakeholders at the organizational level (e.g. management of OHS), the work environment (e.g. employers, HR management), and other health care professionals to addressing the conditional external barriers for guideline use and organisational constraints.

In addition to work-contextual constraints OPs perceived other external barriers related to the guideline itself. OPs perceived the guideline as being unclear and inconsistent, with complex terminology [29]. These factors in particular need to be changed by guideline developers in order to help professionals use the guideline in their daily practice. At present the Netherlands Society of Occupational Medicine is revising the guideline which is expected to be released in 2019 [51].

### Strengths and limitations

A strength of this study is its randomized controlled design, which is rare in this field of research [52]. By using cluster randomization the risk of contamination between the intervention and control condition was low. Another strengths is that we evaluated the use of the guideline in daily practice in two groups of OPs after completing 1 year of guideline training. The risk for recording desired performance by the OP is minimal, since the data collection started 3.5 year after the OPs gave their consent. In addition, by means of a conscientious and thorough development process a new set of PIs was developed for the revised version of the practice guideline. This may facilitate development and evaluation of international guidelines on this relevant and growing topic.

Assessing guideline adherence by an audit of medical files is, as with any observational study, susceptible to bias. A possible source of bias is that physicians did not document all their findings systematically in the medical records, resulting in an underestimation of the true performance. However, the method of auditing medical records is also a strength, since it hardly interferes with actual performance, in contrast to actual or video observation of consultations. Moreover, it is found that objective measures of adherence such as medical records are more accurate than self-perceived adherence which tend to result in an overestimation of adherence [53]. Another limitation is that the performance indicators might not influence guideline adherence in an equal way, that is, some performance indicators might have been conditional for others. For example, if an OP does not have regular contact with the employer (PI5.3), he/she will presumably report less information about the perspective of the employer regarding the recovery of the worker (PI1.3). To prevent interpretation bias, all medical records were blindly assessed by two researchers independently and a third researcher in case no consensus was reached. Since this is a pragmatic trial, in which we tested the effectiveness of guideline training in a real life situation, we have used intention to treat analysis [54]. Ideally, an OP guides a worker throughout the entire sickness and recovery process. However, in practice the worker might be allocated to another OP, because of (holiday) leave of the OP, or because the OP changes location. In all cases, the worker’s medical record was analyzed in the way the worker was randomized at the beginning of the trial, regardless of whether the worker completed their guidance with the same OP. By using this approach type II errors may occur and this should be taken into account when translating the results to another setting. Another limitation is the small sample size achieved; from 22 out of 56 participating OPs medical recordings were not assessed because none of the workers guided by these OPs were included in the study, which might have caused selection bias. Unfortunately, no information was available from workers who did not participate in the study, for which reason a non-response analysis could not be conducted. However, no significant differences were found between workers characteristics in the control and intervention group. In addition, using the GLMM analyses showed that adding OP as a random effect did not significantly improve the model.

## Conclusion

The results from this study support the idea that a tailored implementation strategy in small interactive peer sessions during a long interval is effective in implementing guidelines but has limited impact if external barriers continue to hinder guideline adherence. We found that peer-group guideline training, focusing on perceived barriers, improved adherence to the guideline for mental health problems in occupational health care. As a generic approach to address key barriers for change, the implementation strategy might also be an effective method for implementing other guidelines, in other health care professionals and/or in other countries. To optimize the implementation process of guidelines, future research should focus on the implementation of interventions that target different levels (provider level, patient/worker level and organizational level), and should involve relevant stakeholders who are committed to implementing guideline recommendations, such as OPs, management of occupational health services, employers, workers and other health care professionals.

## Additional file

Additional file 1: Example of the implementation of a guideline recommendation by OPs participating in the guideline training’ an example is presented of how OPs were engaged in the implementation of the guideline recommendations and how a PDCA cycle was applied. (DOCX 64 kb)

## Abbreviations

4DSQ: Four Dimensional Symptom Questionnaire (4DKL in Dutch)
GLMM: Generalized linear mixed models
MHP: Mental health problems
NVAB: Netherlands Society of Occupational Physicians (NVAB in Dutch)
OHS(s): Occupational health service(s)
OP(s): Occupational physician(s)
PDCA: Plan-do-check-act
PI(s): Performance indicator(s)
RTW: Return-to-work
WAI: Workability index
