# Supplementary material for: Effectiveness of a tailored implementation strategy to improve adherence to a guideline on mental health problems in occupational health care
Source: BMC Health Serv Res. 2019 May 3;19:281. doi: 10.1186/s12913-019-4058-5 (PMC6499945; doi:10.1186/s12913-019-4058-5)
Supplement: Supplementary file 1 — Example of the implementation of a guideline recommendation by OPs participating in the guideline training’ an example is presented of how OPs were engaged in the implementation of the guideline recommendations and how a PDCA cycle was applied. (DOCX 64 kb) [file 12913_2019_4058_MOESM1_ESM.docx]

**Additional file 1. Example of the implementation of a guideline recommendation by OPs participating in the guideline training**

In this example a specific guideline recommendation is used namely ‘use the Four Dimensional Symptom Questionnaire (4DSQ) for diagnostic purposes, for monitoring symptoms and for providing feedback to the patient and/or other caregivers’.

Studying guideline text

Creation of an electronic 4DSQ tool 🡪 trainer disseminates electronic 4DSQ tool among OPs

TEST SOLUTIONS

Case discussion (i.e. next meeting cases are discussed)

PERCEIVED BARRIERS

GROUP DISCUSSION

Lack of knowledge/understanding of the 4DSQ

Limit access paper version of the 4DSQ (not available at the work site)

- Dissemination of guideline recommendation and information by the trainer on the purpose of the 4DSQ

- Discussing the guideline recommendation in peer group

MEETING 1

OPs test solutions in daily practice

SOLUTIONS

Negative attitude towards use 4DSQ (i.e. not useful in practice)

Lack of time / not used to work with 4DSQ

Case discussion (i.e. next meeting cases are discussed)

No further actions are needed

Individual guidance from peers

TEST SOLUTIONS

Share practical tips with peers from other groups (via the trainer)

PERCEIVED BARRIERS

GROUP DISCUSSION

*Solved barriers*: Knowledge of the purpose of 4DSQ

*Remaining barrier*: Lack of time; Lack of belief that 4DSQ is useful

- Discuss experiences with (peer) group members

- Case discussion: one OP introduces case and receives feedback from peers

MEETING 2

OPs test solutions in daily practice

SOLUTIONS

*New barriers*: Lack of skills and confidence to use 4DSQ; Difficult to adopt new assessment strategy

No further actions are needed

Individual case discussion

TEST SOLUTIONS

Use 4DSQ specifically to monitor symptoms over time

PERCEIVED BARRIERS

GROUP DISCUSSION

*Solved barriers*: For most OPs the electronic 4DSQ is a feasible tool; Belief that 4DSQ is an useful tool

*Remaining barrier*: Lack of skills and confidence to use 4DSQ; Difficult to adopt new assessment strategy; Lack of time

- Discuss experiences with (peer) group members

- Share good practices (within group and between groups)

MEETING 3

OPs test solutions in daily practice

SOLUTIONS

*New barriers*: OPs make limited use of 4DSQ to monitor symptoms; Difficult to apply to suicidal patients

No further actions are needed

Keep evaluating the use of 4DSQ with peers

TEST SOLUTIONS

Keep evaluating the use of 4DSQ with peers

PERCEIVED BARRIERS

GROUP DISCUSSION

*Solved barriers*: OPs are skilled and confident to use 4DSQ; OPs feel empowered when using 4DSQ (e.g. to discuss ‘difficult’ topics with patient, in contact with other health care providers)

*Remaining barrier*: Lack of time due to workload; takes time to fully adopt a new assessment strategy

- Discuss experiences with (peer) group members

- Individual case discussion (provide feedback to peer’s case)

MEETING 4

OPs test solutions in daily practice

SOLUTIONS

*New barriers*: Lack of skills to interpret results from 4DSQ as monitor

Source: Joosen et al. (2015). Improving occupational physicians' adherence to a practice guideline: feasibility and impact of a tailored implementation strategy. BMC Medical Education, 15:82.
